# The median effective dose of esketamine with different doses of oliceridine during hysteroscopic surgery

**DOI:** 10.3389/fphar.2025.1728802

**Published:** 2025-12-01

**Authors:** Min Shi, Tong Wang, Weiwei Wang, Jiafeng Wang, Lulu Li, Lulong Bo, Xiaolin Wang

**Affiliations:** Department of Anesthesiology, Changhai Hospital, Naval Medical University, Shanghai, China

**Keywords:** anesthetics, oliceridine, esketamine, 50% effective dose, hysteroscopic surgery

## Abstract

**Purpose:**

This study aimed to investigate the impact of different doses of oliceridine on the ED_50_ of esketamine during hysteroscopic surgery. The objective was to establish an optimal dosing regimen that facilitates the development of an effective and safe analgesic strategy for this procedure by leveraging the potential synergistic effects between the two drugs.

**Methods:**

The trial was conducted involving 90 patients scheduled for elective hysteroscopy. Participants were allocated into three groups: control (0 mg oliceridine), group O1 (1 mg oliceridine), and group O2 (2 mg oliceridine). Anesthesia was induced with propofol, followed by a continuous infusion of propofol and a preset dose of esketamine. The primary outcome was the ED_50_ of esketamine, determined using Dixon’s up-and-down method. Secondary outcomes included recovery time, hemodynamic parameters, pain and sedation scores, and the incidence of adverse events.

**Results:**

The ED_50_ of esketamine was 0.76(0.66–0.86), 0.45(0.40–0.55), and 0.41 (0.31–0.59) mg/kg/h in the control, group O1 and O2, respectively. Compared with the control, group O1(*P* = 0.020) and O2(*P* = 0.001) showed significantly shorter recovery time. Hemodynamic stability was comparable across groups, though the effect on HR was observed: bradycardia incidence was higher in group O1 than in the Control (*P* = 0.021) but lower in group O2 than in O1(*P* = 0.004). Compared to the control, the O1 and O2 groups showed a significantly reduced incidence of both excessive oral secretion (3.7% in group O2 vs. 0.0% in group O1 vs. 32.0% in control, *P* = 0.000) and cough (4.3% in group O1 and 0.0% in group O2 vs. 28.0% in the control, *P* = 0.002). The combination therapy did not increase respiratory adverse reactions (*P* > 0.05), and the 2 mg of oliceridine appeared to provide optimal balance between efficacy and safety within the limits of this study.

**Conclusion:**

For hysteroscopic procedures, the co-administration of oliceridine was associated with a lower ED_50_ of esketamine. This regimen provided synergistic analgesia, reducing the ED_50_ of esketamine to lower deep sedation and accelerate recovery. Furthermore, it improved hemodynamic stability by lowering bradycardia incidence without augmenting respiratory adverse effects.

**Trial Registration:**

www.chictr.org.cn, (ChiCTR2500101056); registration date: April 18, 2025.

## 1 Introduction

Hysteroscopy has become a well-established approach for the diagnosis and treatment of gynecological disorders due to its minimally invasive nature, technical simplicity, and rapid postoperative recovery (Pan et al., 2024). Nevertheless, intraoperative maneuvers such as cervical dilation and endometrial intervention can provoke pain, which may lead to anxiety, stress responses, and even procedural interruption (Lan et al., 2023). Although propofol-opioid regimens remain the standard anesthetic approach, conventional opioids often cause respiratory depression and gastrointestinal side effects through β-arrestin pathway activation, thus restricting their use in clinical procedures (Smith et al., 2019).

Oliceridine, a novel μ-opioid receptor agonist, has demonstrated potent analgesic efficacy with a reduced activation of β-arrestin pathway (Ke et al., 2025). Meanwhile, esketamine, an N-methyl-D-aspartate (NMDA) receptor antagonist, provides effective sedation and analgesia while maintaining respiratory and hemodynamic stability (De Sloovere et al., 2025), but its utility at higher doses is limited by dose-related neuropsychiatric effects, including dizziness and profound sedation (Zhong et al., 2023; Gastaldon et al., 2021; Luo et al., 2024). Interestingly, the study by Sarasso et al. (2024) demonstrated that dissociation, often misinterpreted as an adverse effect of esketamine, in fact exerted therapeutic actions in specific depressive subtypes, particularly in treatment-resistant depression (Di et al., 2024). Therefore, the pharmacodynamics of esketamine are multifaceted and complex, with its therapeutic potential and complex psychotropic properties warranting further exploration.

Rational combination of oliceridine and esketamine may yield synergistic analgesic benefits while mitigating individual drug–specific adverse effects (Huang et al., 2024; Sałat et al., 2021). However, the specific impact of oliceridine on the median effective dose (ED_50_) of esketamine during hysteroscopy remains unquantified. No relevant trials have examined whether concomitant oliceridine reduces esketamine requirements, maintaining hemodynamic stability.

Therefore, the study was designed to investigate the impact of various oliceridine doses on the ED_50_ of esketamine during hysteroscopy. Our objective is to establish an effective dosing regimen that facilitates the development of analgesic strategy for hysteroscopy.

## 2 Methods

### 2.1 Ethical approval and trial registration

This randomized controlled study was endorsed by Shanghai Changhai Hospital Medical Ethics Committee (approval number: CHEC2025-117) and was also registered in Chinese Clinical Trial Registry prior to its start (registration number: ChiCTR2500101056; registration date: 04/18/2025). The procedures carried out in this study adhered to the ethical standards of research and the Declaration of Helsinki. Informed consent was obtained from all participating patients prior to enrollment.

### 2.2 Criteria for inclusion and exclusion

A prospective randomized controlled study was conducted at Shanghai Changhai Hospital. From May 2025 to August 2025, patients scheduled for elective hysteroscopy under intravenous anesthesia were eligible for participation in this trial if they: (i) were 30–65 years old; (ii) were with body mass index (BMI) between 18 and 28 kg/m^2^; (iii) had American Society of Anesthesiologists (ASA) physical status classification of I–II; and (iv) were able to sign an informed consent form. Patients were excluded when they had: (i) a history of severe cardiovascular, cerebrovascular, pulmonary, hepatic, renal, or metabolic diseases; (ii) hypertension (systolic blood pressure>180 mmHg) or hypotension (systolic blood pressure<90 mmHg); (iii) thyroid dysfunction; (iv) mental disorders, long-term psychiatric medication treatment, and cognitive impairments (Sarasso et al., 2024); (v) heart rate (HR) < 55 bpm or high-degree atrioventricular block requiring pacemaker use; (vi) severe respiratory diseases (obstructive sleep apnea, acute respiratory tract infection, chronic obstructive pulmonary disease, asthma, etc.); (vii) been known allergy to emulsions or opioid drugs; (viii) surgical duration>1 h; (ix) emergency surgery.

### 2.3 Preoperative arrangements and anesthesia procedure

The patients were randomized into 3 groups according to the allocation plan inside the sealed envelopes: control (0 mg oliceridine); group O1 (1 mg oliceridine); and group O2 (2 mg oliceridine). The dose of oliceridine was selected based on the study by Brzezinski et al. (2021). The random allocation sequence was generated by an independent statistician, using computer-generated simple randomization. Allocation concealment was achieved through sequentially numbered, opaque, sealed envelopes, which were prepared by a researcher. The researchers who performed the randomization and blinding procedure did not participate in the follow-up study. Both the participants and anesthesiologists were blinded to the group assignments.

All participants were required to fast from food for 8 h and from liquids for 6 h before surgery. They also refrained from any preoperative medications. The patients were established by a 20-gauge peripheral intravenous catheter for Ringer Lactate solution infusion and drugs. Concurrently, we maintained continuous monitoring of the electrocardiogram, oxygen saturation, and non-invasive blood pressure (NIBP). During the study, oxygen was delivered via a nasal catheter at a flow rate of 3–5 L/min. Microstream, ETCO_2_ monitoring was performed via the nasal catheter to measure partial pressure of end-tidal CO_2_ (PetCO_2_) levels.

Following preoxygenation, control, group O1and group O2 received no oliceridine,1 mg oliceridine, and 2 mg oliceridine, respectively. The loading dose of propofol 1–1.5 mg/kg was administered before perineal disinfection. Subsequently, a continuous intravenous infusion of propofol was sustained at a rate of 4–6 mg/(kg.h). While infusing propofol, esketamine was initiated at the preset dose, with the concentration of 1 mg/mL. The initial infusion rate of esketamine was 1 mg/kg/h in control, 0.3 mg/kg/h in group O1, and 0.36 mg/kg/h in group O2 (Schwenk et al., 2018). The hysteroscopic procedure was initiated when the eyelash reflex was absent and the modified observer’s assessment of alertness/sedation score (MOAA/S) was 0–1. In cases where adequate sedation was not achieved, an supplementary dose of propofol (0.5 mg/kg) was administered. In the event of insufficient analgesia, additional doses of esketamine(0.05 mg/kg) was administered as required. An MOAA/S score of five indicates that the patient is fully awake and responds readily when their name is called in a normal tone; an MOAA/S score of 0 signifies that the patient does not respond to a noxious stimulus (Cheng et al., 2024).

In accordance with Dixon’s up-and-down method, the dosage of esketamine for the ensuing patient was adjusted upon the preceding patient’s response. Any physical movement in response to cervical dilation was considered as a “negative response”, otherwise it was considered as a “positive response”. If the patient failed, the next patient received an increased dose based on the original dose; conversely, if the patient succeeded, the next patient received a reduced dose. The dose of esketamine ratio between two consecutive patients is 1:1.2 (Zheng et al., 2022). The formal test commences with the first positive/negative pairs, and continues until at least the seventh positive/negative pairs, which is considered as a termination point. During the operation, in the event of bradycardia, 0.5 mg of atropine is administered. When systolic blood pressure (SBP) is less than 90 mmHg or mean arterial pressure (MAP) is less than 60–65 mmHg, ephedrine or phenylephrine is given based on clinical experience. If pulse oximetry (SpO_2_) drops below 90%, the oxygen flow rate is heightened and the airway is opened by performing a jaw thrust maneuver. Assisted ventilation is provided if necessary.

### 2.4 Measurements

#### 2.4.1 Primary outcome

The ED_50_ of esketamine combined with oliceridine for intravenous anesthesia in hysteroscopic surgery was determined.

#### 2.4.2 Secondary outcome

The researchers collected the data including demographic information, intraoperative vital signs, recovery-related parameters and adverse events. SBP, diastolic blood pressure (DBP), MAP, HR, SpO_2_ and PetCO_2_ were obtained at the specified time points. At the specified time points: T1, entry to operation room; T2, sedation induction; T3, 1 min after induction; T4, cervical dilation; T5, 2 min post-hysteroscope; T6, surgery end; T7, exit operation room; T8, enter post-anesthesia care unit (PACU); T9, exit PACU.

Perioperative anesthesia-related adverse events, such as respiratory depression, hypoxia, hypotension, hypertension, bradycardia, nausea and vomiting, dizziness, somnolence, excessive oral secretion, cough and mental symptom were recorded. The recovery time is defined as the duration from the cessation of anesthetic administration to the point when the patients awoke with eyes open. The Numerical Rating Scale (NRS) serves as a prevalent method for evaluating the severity of pain (Rubio-Zarapuz et al., 2024). Individuals are requested to quantify their pain on a numeric continuum ranging from 0 to 10, with 0 indicating “no pain” and 10 signifying “the most severe pain imaginable”. The patients’ NRS and MOAA/S scores were assessed immediately after awakening and again upon discharge from the PACU and 2 h, 6 h postoperatively. Hypoxia was defined as the SpO_2_ level less than 90% lasting for more than 1 min (Qiu et al., 2022). Recovery time was defined as the time interval from the cessation of anesthetics to eye opening in response to verbal command (Kwon et al., 2016).

### 2.5 Statistical analysis

According to the up-and-down sequential allocation method, approximately 20–40 participants were needed to reliably estimate the ED_50_, with at least seven pairs of sequence reversals being considered adequate (Wan et al., 2024; Pace and Stylianou, 2007). Based on the study by Oron et al. (Oron et al., 2022), we planned to enroll 30 participants per group, with the final enrollment subject to the number of pairs achieved during the study.

Continuous variables were presented as mean ± SD, and categorical variables were depicted as N (%) of patients. All data were tested for normality using the Shapiro-Wilk test. An analysis of the dose-response relationship via probit regression provided an estimate of the ED_50_ and 95% confidence interval (CI). To compare differences among different groups, one-way ANOVA was performed, followed by Bonferroni correction for *post hoc* pairwise comparisons. For the assessment of continuous variables at multiple time points within each group, a two-way repeated-measures ANOVA was performed. Subsequently, simple effects analysis was carried out to explore potential interaction effects between group and time. In the case of categorical variables, the Pearson chi-square test or Fisher’s exact test was applied as appropriate. Statistical analyses were conducted using SPSS 25.0(IBM, United States) or GraphPad Prism 6.0 (GraphPad Software, United States). A *P* value less than 0.05 was set to determine statistical significance.

## 3 Results

**FIGURE 1:**
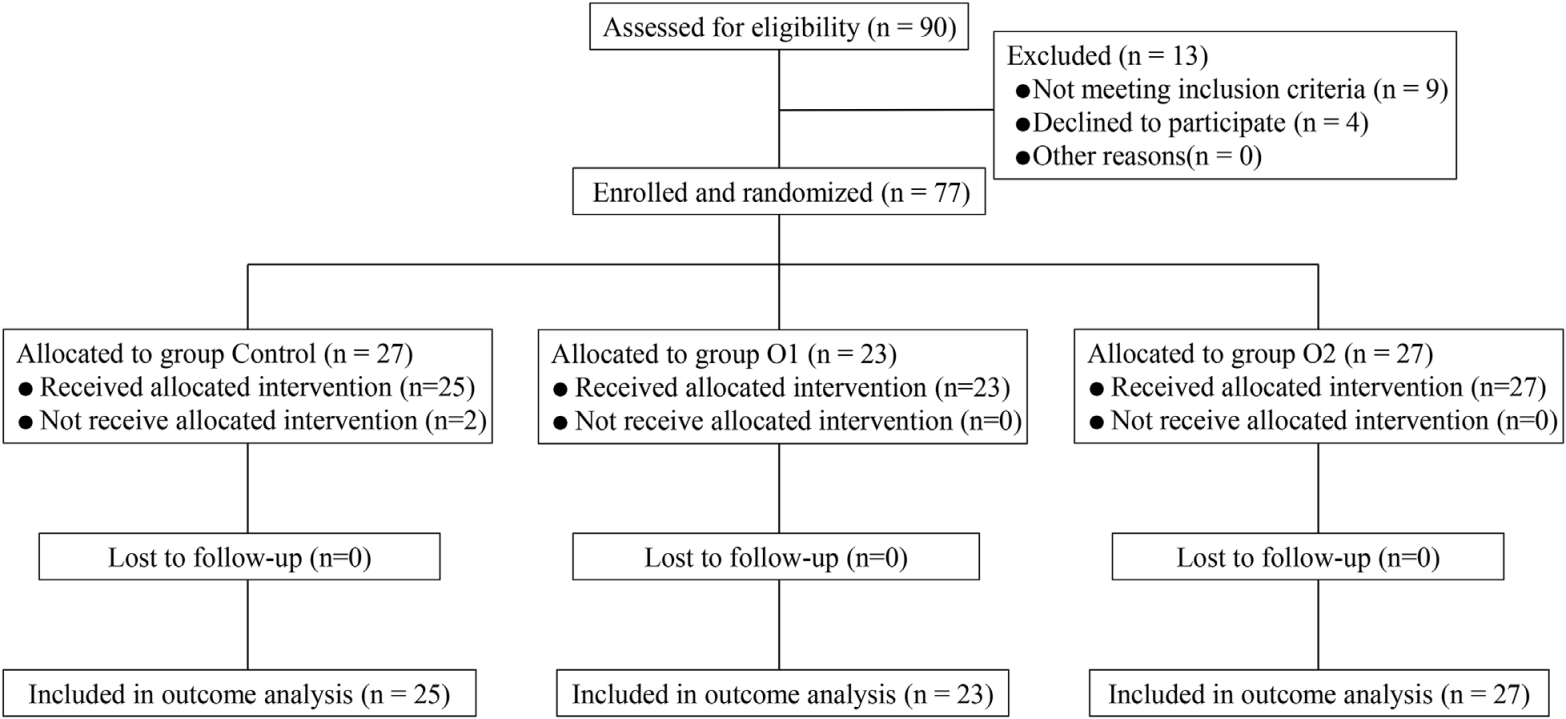
Study flow diagram.

90 patients were recruited altogether in this study. Several exclusions led to the need for adjustments: Nine patients failed to meet the inclusion criteria, four patients declined participation, and two patients were removed due to the conversion to general anesthesia intraoperatively., as presented in Figure 1.

### 3.1 Demographics and clinical features

There were no statistical differences in age, BMI, ASA classification, chronic disease, alcohol consumption, allergic history, surgical history, and operation time among the three groups, suggesting comparable baseline characteristics (Table 1).

**TABLE 1:** Demographic data and clinical characteristics in three groups. Data are presented as mean ± SD or n (%). ASA, American Society of Anesthesiologists; BMI, body mass index.

Items	Control (n = 25)	Group O1 (n = 23)	Group O2 (n = 27)	p value
Age(y)	45.1 ± 9.8	43.8 ± 10.2	44.3 ± 7.5	0.885
BMI(kg/m^2^)	22.3 ± 2.4	22.8 ± 2.9	23.1 ± 3.0	0.577
ASA				0.597
I	12 (48.0)	14 (60.9)	13 (48.1)	
II	13 (52.0)	9 (39.1)	14 (51.9)	
Chronic disease				
Hypertension	3 (12.0)	4 (17.4)	4 (14.8)	0.870
Hypothyroidism	1 (4.0)	0	2 (7.4)	0.412
Arrhythmia	1 (4.0)	3 (13.0)	1 (3.7)	0.338
Motion sickness	1 (4.0)	0	0	0.363
Others	1 (4.0)	2 (8.7)	1 (3.7)	0.689
Alcohol consumption	0	1 (4.4)	0	0.318
Allergic history	4 (16.0)	3 (13.0)	2 (7.4)	0.624
Surgical history	17 (68.0)	17 (73.9)	18 (66.7)	0.844
Duration of operation (min)	26.7 ± 10.8	23.4 ± 9.2	26.6 ± 11.3	0.478

### 3.2 Median effective dose

**FIGURE 2:**
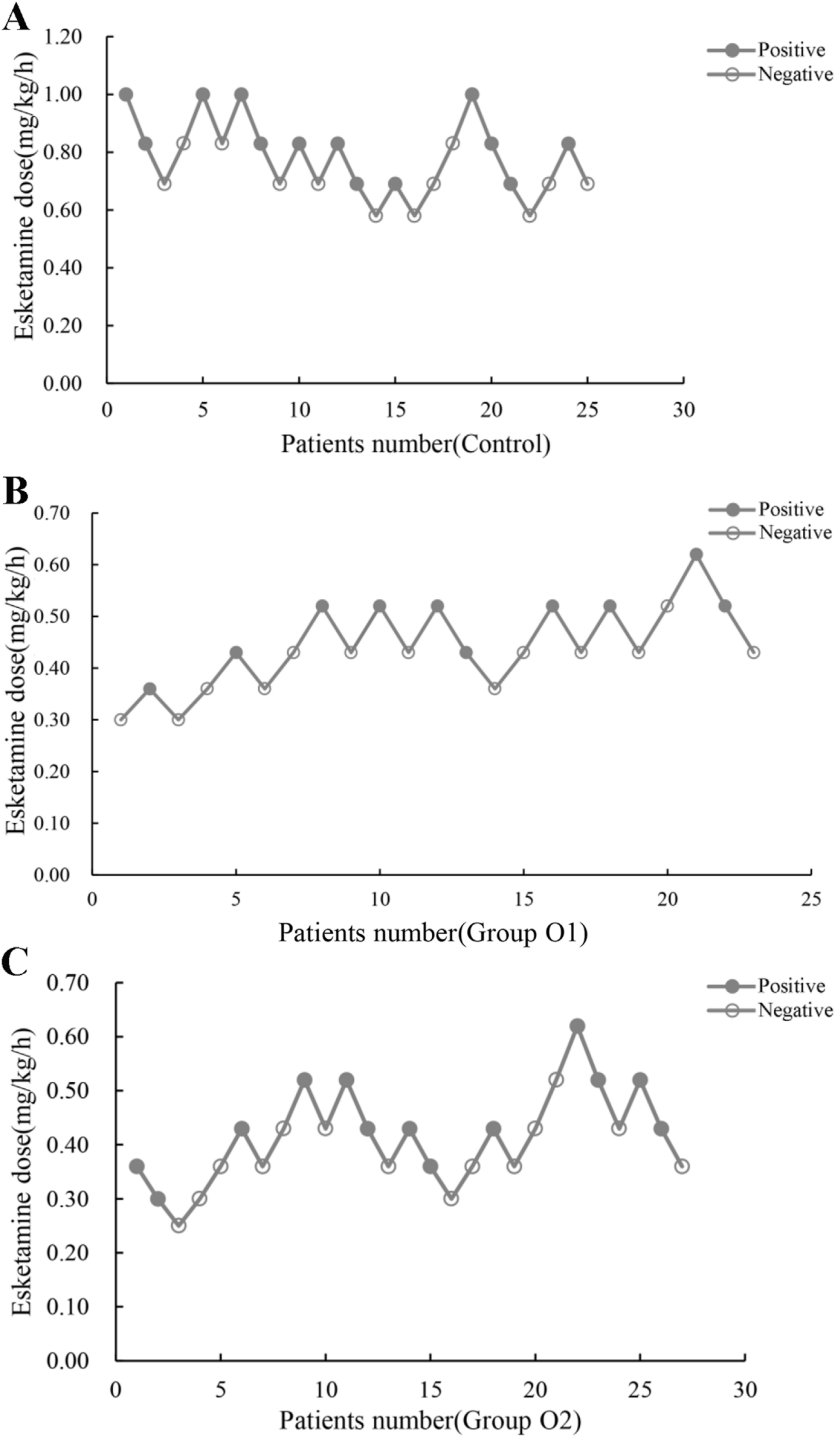
Individual reactions to esketamine at corresponding dose (mg/(kg.h)). **(A)** Control: 0 mg oliceridine combined with esketamine; **(B)** Group O1: 1 mg oliceridine combined with esketamine; **(C)** Group O2: 2 mg oliceridine combined with esketamine.

Esketamine ED_50_ and its 95% CI for hysteroscopic surgery were, 0.76 (0.66–0.86), 0.45 (0.40–0.55) and 0.41 (0.31–0.59) mg/kg/h in control, group O1 and group O2, respectively (Table 2). The probability unit regression equation fitted for control, group O1 and group O2 based on probit regression analysis was: probit(P) = 1.813 + 14.952×lg (esketamine dose), probit(P) = 4.526 + 13.214×lg (esketamine dose), and probit(P) = 2.916 + 7.532×lg (esketamine dose), respectively. Compared with control, ED_50_ of esketamine in group O1 and group O2 decreased by 40.8% and 46.1%. The ED_50_ of esketamine differed among the groups, as shown in Figure 2.

**TABLE 2:** Esketamine ED_50_ and its 95% CI.

Items	Control (n = 25)	Group O1 (n = 23)	Group O2 (n = 27)
Esketamine ED50 (mg/kg/h)	0.76	0.45	0.41
95% CI	0.66, 0.86	0.40, 0.55	0.31, 0.59

### 3.3 Recovery time

**FIGURE 3:**
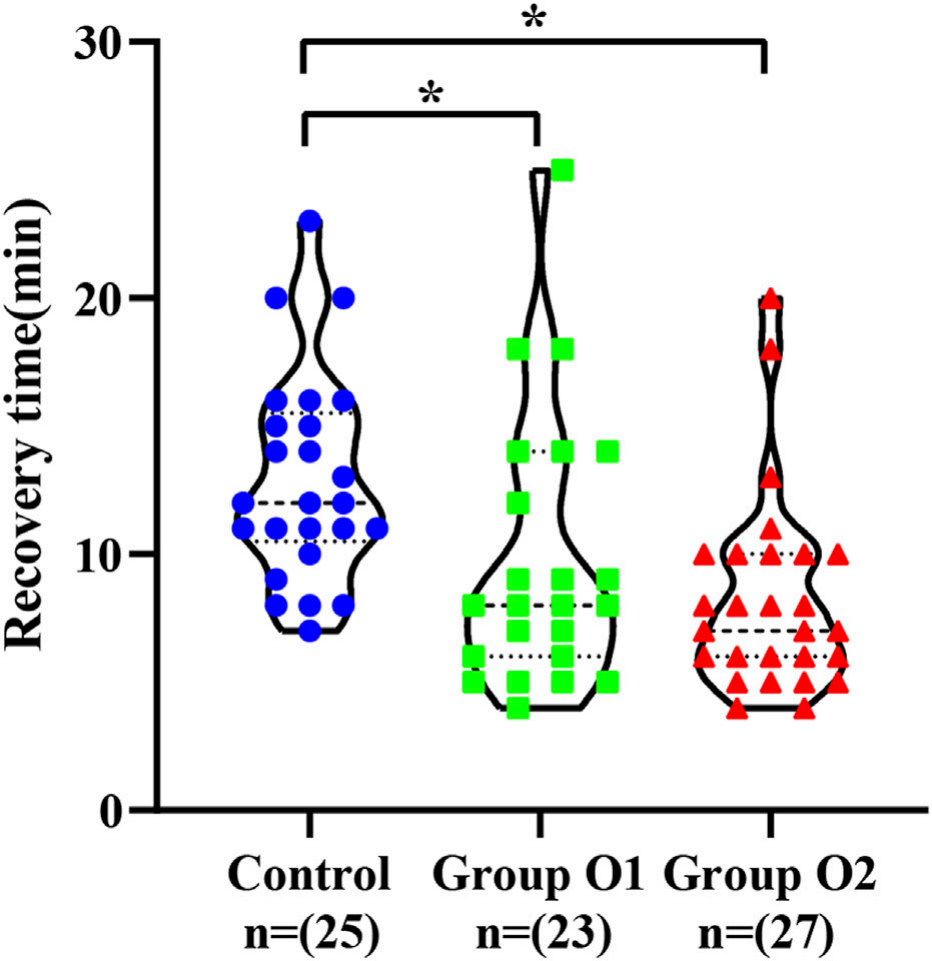
Violin plots showed the distribution of recovery time in control, group O1 and group O2. (*P < 0.05 vs. Control).

The recovery time for the three groups were 12.9 ± 4.1 min in the control, 9.7 ± 5.3 min in group O1, and 8.3 ± 3.9 min in group O2, respectively, as shown in Figure 3. Compared with control, the awakening time in group O1 (95% CI: -5.90, −0.47; *P* = 0.0227) and O2 (95% CI: -6.86, −2.46; *P* < 0.0001) were statistically reduced derived from one-way ANOVA.

### 3.4 Hemodynamic characteristics

Significant effects of time were observed for the parameters of SBP, DBP, MAP, HR, and PetCO_2_, *P* = 0.000), indicating substantial fluctuations throughout the period. The group effects were not significant for any parameter (all *P* > 0.05), suggesting comparable overall hemodynamic levels among three groups, as shown in Table 3.

**TABLE 3:** Results of repeated-measures ANOVA for hemodynamic parameters. All time and interaction effects were adjusted using Greenhouse-Geisser correction when sphericity was violated. SBP, systolic blood pressure; DBP, diastolic blood pressure; MAP, mean arterial pressure; HR, heart rate; PetCO_2_, partial pressure of end-tidal carbon dioxide; SpO_2_, pulse oximetry.

Hemodynamic parameter	Group effect (p value)	Time effect (p value)	Group × Time interaction (p value)
SBP	0.955	0.000	0.006
DBP	0.573	0.000	0.011
MAP	0.653	0.000	0.052
HR	0.303	0.000	0.098
PetCO_2_	0.598	0.000	0.584
SpO_2_	0.421	0.196	0.279

Notably, significant Group × Time interactions were found for SBP (*P* = 0.006) and DBP (*P* = 0.011), demonstrating differential temporal patterns across group O1 and O2. However, no significant interactions were observed for MAP, HR, PetCO_2_, or SpO_2_ (all *P* > 0.05). Detailed values for all indicators are provided in Supplementary Figure S1.

### 3.5 Numerical rating scale and modified objective awareness scale/sedation assessment scale

We performed NRS and MOAA/S scores at three time points of in PACU, 2 h postoperatively and 6 h postoperatively. There was no statistically significant difference in NRS and MOAA/S scores among the three groups (*P* > 0.05), suggesting a similar effect overall, as illustrated in Table 4.

**TABLE 4:** Postoperative pain and sedation score. Data are presented as mean ± SD. NRS, Numeric Rating Scale; MOAA/S, Modified Objective Awareness Scale/Sedation Assessment Scale.

Items	Control (n = 25)	Group O1 (n = 23)	Group O2 (n = 27)	p value
PACU				
NRS	1.7 ± 2.1	2.5 ± 1.9	1.2 ± 1.5	0.059
MOAA/S	4.3 ± 0.7	4.6 ± 0.7	4.6 ± 0.7	0.143
2 h postoperatively				
NRS	1.4 ± 1.8	1.6 ± 1.8	0.8 ± 1.2	0.172
MOAA/S	4.7 ± 0.5	4.8 ± 0.5	5.0 ± 0.2	0.093
6 h postoperatively				
NRS	0.2 ± 0.7	0.5 ± 1.1	0.1 ± 0.3	0.154
MOAA/S	5.0 ± 0.0	5.0 ± 0.0	5.0 ± 0.0	—

### 3.6 Side effects

The incidence of respiratory depression, hypoxia, hypotension, hypertension, nausea and vomiting, dizziness, somnolence, and mental symptom indicated no significant differences among the three groups (*P* > 0.05). However, the incidence of both excessive oral secretions (control: 32.0%; O1: 0.0%; O2: 3.7%) and cough (control: 28.0%; O1: 4.3%; O2: 0.0%) was significantly reduced in the O1 and O2 groups compared to the control group (*P* = 0.000 for secretions; *P* = 0.002 for cough). Notably, the incidence of bradycardia varied among the three groups; in contrast to control, the incidence was significantly higher in group O1 (*P* = 0.021); compared with group O1, group O2 had a significantly lower incidence (*P* = 0.004). All findings were presented in the Table 5.

**TABLE 5:** Adverse events. Data are presented as n (%). (*P < 0.05 vs. Control; ^#^ *P* < 0.05 vs. Group O1).

Items	Control (n = 25)	Group O1 (n = 23)	Group O2 (n = 27)	p value
Respiratory depression	4 (16.0)	2 (8.7)	10 (37.0)	0.614
Hypoxia	0	0	4 (14.8)	0.166
Hypotension	0	2 (8.7)	0	0.100
Hypertension	2 (8.0)	0	0	0.132
Bradycardia	1 (4.0)	5 (21.7)*	0^#^	0.011
Nausea and vomiting	4 (16.0)	4 (17.4)	4 (14.8)	0.971
Dizziness	3 (12.0)	3 (13.0)	3 (11.1)	0.979
Somnolence	8 (32.0)	3 (13.0)	6 (22.2)	0.301
Excessive oral secretion	8 (32.0)	0*	1 (3.7)*	0.000
Cough	7 (28.0)	1 (4.3)*	0*	0.002
Mental symptom	2 (8.0)	0	0	0.132

## 4 Discussion

The results of the study indicated that oliceridine was associated with a reduction in the ED_50_ of esketamine. Compared with control, group O1 and O2 exhibited reductions in the ED_50_ of esketamine by 40.8% and 46.1%, respectively. Furthermore, patients treated with oliceridine exhibited a significantly shorter recovery time, without an increase in adverse reactions such as respiratory depression and hypoxemia. It was noteworthy that the incidence of bradycardia was significantly higher in group O1 compared to the control, while the incidence in group O2 was significantly lower than that in group O1, suggesting a potential non-linear trend of oliceridine on HR requiring further investigation. Therefore, 2 mg of oliceridine appeared to provide optimal balance between efficacy and safety within the limits of this study. In addition, no additional adverse events were observed due to the small sample size.

The purpose of this study was to reduce the dosage of esketamine through a combination drug strategy, primarily to mitigate the potential risks associated with deep sedation. Esketamine, although causing mild respiratory depression, is often associated with a range of adverse reactions when it reaches the state of deep sedation (Dijkstra et al., 2022). Firstly, neuropsychiatric symptoms, such as dizziness, hallucinations, abnormal dreams, and delirium, are its common toxicities, which rises significantly at higher plasma concentrations. These effects severely impact the quality of patients’ emergence from anesthesia and postoperative experience, and prolong the duration of monitoring in the PACU. Secondly, Yang et al. (2022) confirmed that esketamine’s inherent sympathomimetic properties may be counteracted by the cardiovascular-depressing effects of concomitant sedatives such as propofol, potentially leading to hypotension or bradycardia. Furthermore, deep sedation compromised airway patency, predisposing patients to complications such as upper airway obstruction from tongue retraction due to muscular atonia (Shao et al., 2020). Moreover, even when SpO_2_ is maintained by simple maneuvers, the increased workload and risk of hypoxia remain. Consequently, it is significant to explore strategies to reduce the clinical dosage of esketamine.

Oliceridine, a novel G protein-biased μ-opioid receptor agonist, possesses a mechanism of action fundamentally distinct from that of conventional opioids (Viscusi et al., 2019). Beyond activating the G-protein pathway to produce analgesia, it is also focused on minimizing β-arrestin pathway activation (Mallo-Abreu et al., 2021). The activation of β-arrestin pathway is established as a key mechanism that accounts for the respiratory depression and gastrointestinal complications associated with traditional opioids (Mallo-Abreu et al., 2021). Consequently, oliceridine not only provides good analgesic effects but also has fewer side effects. The findings demonstrated that oliceridine along with esketamine reduces the ED_50_ of esketamine by leveraging synergistic analgesia: esketamine suppresses central sensitization and hyperalgesia via NMDA receptor antagonism, whereas oliceridine activates G-protein signaling to enhance descending inhibitory pathways. This multi-target synergy (Sałat et al., 2021) permits a substantial reduction in the esketamine, thereby avoiding relevant risks induced by excessive esketamine.

The study observed a significantly shorter recovery time in group O1 and O2, which may be attributed to the following factors. Firstly, reduced esketamine consumption. This may be the most direct cause. As shown in the results, the ED_50_ of esketamine was significantly lower when combing oliceridine. A lower infusion rate resulted in a reduced drug load, facilitating a faster recovery from sedation. Secondly, unique pharmacokinetic profile of oliceridine. Oliceridine is characterized by a short distribution half-life and rapid clearance (Hill and DeBoer, 2023). Consequently, plasma concentrations declined after discontinuation, leading to a decrease in mu-opioid receptor occupancy and thereby reducing sustained inhibition of arousal. Lastlioptimized anesthesia depth management. The analgesic foundation provided by oliceridine reduced the reliance on high-dose esketamine, which simultaneously induced deep sedation, thus facilitating rapid emergence.

Strikingly, this study revealed no significant differences in blood pressure, PetCO_2_ or SpO_2_ among the groups. A distinct pattern was, however, observed in HR: the incidence of bradycardia was higher in group O1 compared to group O2. This non-linear dose-response relationship may be explained by the following mechanisms. First, vagus nerve excitation hypothesis. At the dose of 1 mg, oliceridine may slightly enhance vagal tone by activating μ-opioid receptors in brainstem regions such as the nucleus tractus solitarius, leading to a decrease in HR (Henderson et al., 2002). This effect is more pronounced in some patients, consequently resulting in a higher incidence of bradycardia. Second, analgesic advantage at higher dose. At the dose of 2 mg, this superior analgesia more effectively suppresses the stress response (Li et al., 2024) induced by surgical stimuli, such as cervical dilation. The enhanced inhibition of nociceptive stimuli may counteract or mask the mild vagal excitatory effect (Won et al., 2016), thereby resulting in a net clinical manifestation of greater HR stability and a relatively lower incidence of bradycardia. Last, interaction with esketamine. Esketamine itself possesses sympathomimetic properties, which can stimulate the cardiovascular system (Qi et al., 2024). Against the more effective analgesia provided by 2 mg oliceridine, the sympathomimetic effect of esketamine may be more fully expressed, thus helping to prevent an excessive decrease in HR. Regarding adverse effects, the diminished activation of the β-arrestin pathway by oliceridine may result in decreased respiratory depression, thereby enhancing airway safety. Overall, the phenomenon of oliceridine on HR is noteworthy, but its underlying mechanism remains unclear and requires further validation incorporating specific pharmacodynamic measures, such as heart rate variability analysis.

It is noteworthy that the pharmacological profile of esketamine extends well beyond its anesthetic and analgesic properties. Martiadis et al. (2025) highlighted the positive role of adjunctive intranasal esketamine in treatment-resistant depression and revealed differential neurobehavioral responses across genders. This finding suggests that neurobiological and sex-related dimensions should be considered in clinical safety. Although our study primarily focuses on its anesthetic characteristics, these broader considerations hold implications for future research, providing a rationale for clinical translation and personalized interventions.

The study has several limitations, including its limited sample size, single-center design, absence of pharmacokinetic data, and lack of long-term neurocognitive and psychotropic monitoring. Additionally, while Dixon’s sequential method can estimate the median effective dose, it does not capture the complete dose-response curve; thus, clinical application must be cautious. Furthermore, propofol was continuously infused for anesthesia maintenance, and its interaction with oliceridine and esketamine may also have certain impacts (Dai et al., 2022; Jin et al., 2024). Therefore, further studies are needed to explore the mechanisms of propofol and target drugs.

## 5 Conclusion

Within the confines of this study, the co-administration of oliceridine was associated with a lower ED_50_ of esketamine and faster recovery without an increase in adverse events. Future investigations should explore esketamine’s dose-response relationships through combined pharmacodynamic and neurobehavioral assessments.

## Data Availability

The raw data supporting the conclusions of this article will be made available by the authors, without undue reservation.
