# Supplementary figures and images for: The median effective dose of esketamine with different doses of oliceridine during hysteroscopic surgery
Source: Front Pharmacol. 2025 Dec 1;16:1728802. doi: 10.3389/fphar.2025.1728802 (PMC12702736; doi:10.3389/fphar.2025.1728802)

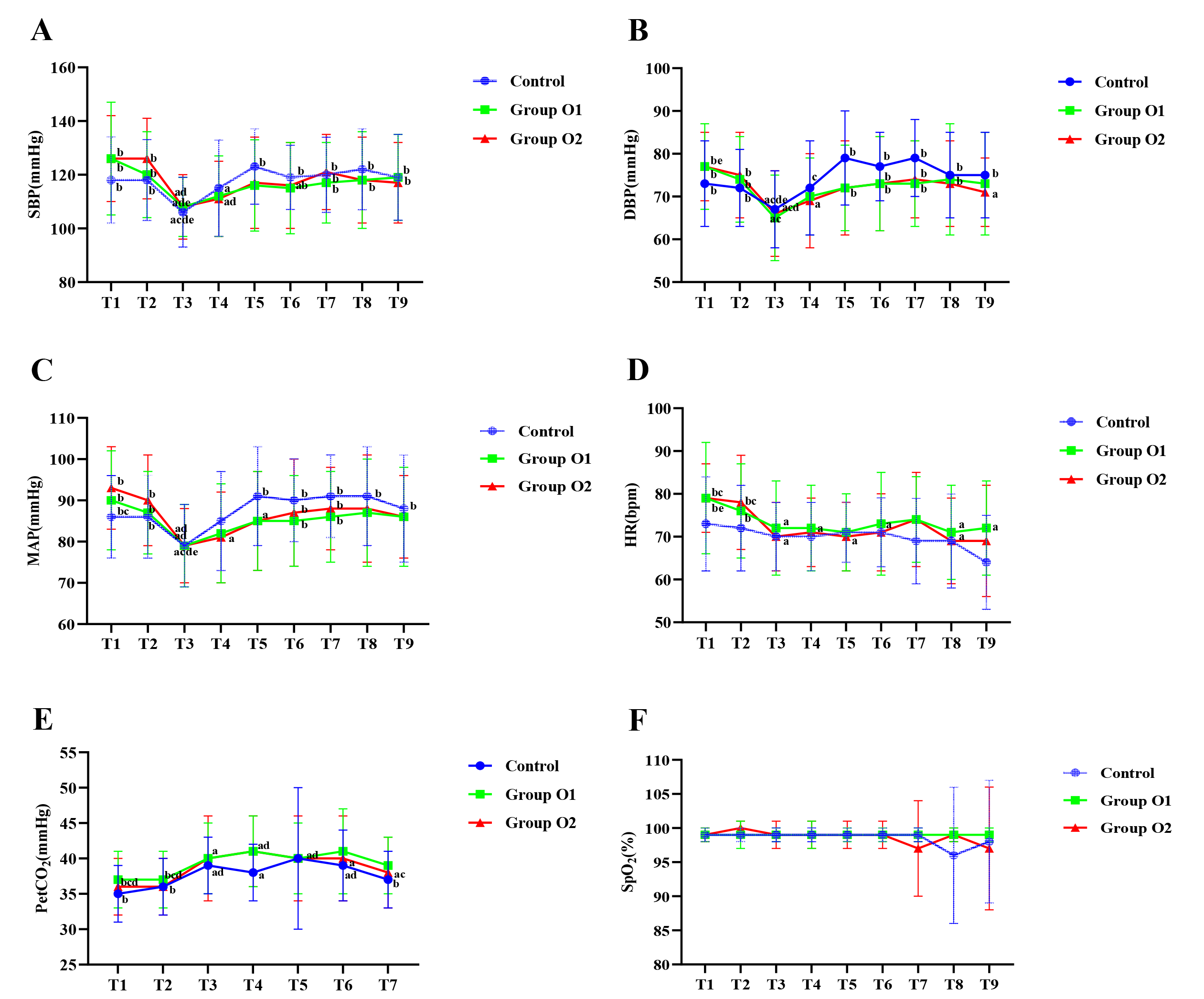

Supplement: Supplementary file 1 [file Image1.tif]
